# Epidemiological characteristics and burden of childhood and adolescent injuries: a survey of elementary and secondary students in Xiamen, China

**DOI:** 10.1186/s12889-015-1726-1

**Published:** 2015-04-10

**Authors:** Ya Fang, Xiang Zhang, Wei Chen, Fang Lin, Manqiong Yuan, Zhi Geng, Hong Yu, Long Dai

**Affiliations:** Key Laboratory of Health Technology Assessment in Fujian Province University, School of Public Health, Xiamen University, Xiamen, China; State Key Laboratory of Molecular Vaccinology and Molecular Diagnostics, School of Public Health, Xiamen University, Xiamen, China; Department of Statistics, School of Economics, Xiamen University, Xiamen, China; Xiamen Institute for Healthcare of Middle and Primary Schools, Xiamen, China; Division of Health Education, Xiamen Center for Disease Control, Xiamen, China

**Keywords:** Injury, Elementary and secondary students, Epidemiology, Burden

## Abstract

**Background:**

Injuries pose a considerable threat to the health of children and adolescents, and childhood injuries cause substantial economic loss for families and society. Many injuries are preventable. To provide a theoretical basis and empirical support for injury prevention interventions, we studied the epidemiological characteristics, risk factors, and burden of injuries among elementary and secondary school students in Xiamen, China.

**Methods:**

Participants were enrolled through multi-stage stratified cluster random sampling of elementary and secondary students in Xiamen in 2010. Questionnaires were completed by students’ parents or guardians to assay students’ basic information, family background, occurrence of injuries in the past year, and burden of injuries. Chi-square tests and logistic regression were performed to identify the key factors of injuries.

**Results:**

A total of 2,816 usable questionnaires reported 365 injury incidents in 303 students over 1 year. The incidence of injuries was 10.8%. Students who were male, extroverted, suburban, had sibling(s), studied in grades 4–9, or whose parents were divorced or separated were more likely to suffer from injuries. Most injuries occurred during the summer months (from June to August), and in the afternoon. The main affected body parts were limbs, fingers or toes. Unintentional falls, collisions/strikes, sprains, and cuts/sharp instrument injuries were the predominant causes of injury. The overall economic burden of the 365 injury incidents was 1,014,649.1 RMB (148,666.5 USD) total, 3,348.7 RMB (490.65 USD) per capita, and 2,779.9 RMB (407.31 USD) per incident.

**Conclusion:**

The injury incidence among elementary and secondary students in Xiamen, China is lower than Guangdong and Zhejiang but higher than Beijing and Shanghai. Injuries caused substantial economic and family burdens and threatened students’ health and life. Childhood and adolescent injuries have become a serious public health problem that requires the urgent attention of the government, society, schools, and families. Injury control and prevention among elementary and secondary school students is essential and will help in multiple ways to reduce the burden on the family to build a harmonious family and society.

## Background

Some injuries require urgent attention and are the leading cause of death for children and adolescents throughout the world [1-5]. According to data published by the World Health Organization (WHO) Global Burden of Disease, millions of children are affected by unintentional injuries annually and more than 2,000 children died from injuries daily in 2004 [2]. Tens of millions of children receive hospital treatment for non-fatal injuries yearly. Moreover, the burden of injury on children is socioeconomically unequal. More than 95% of all injury-caused deaths in children occurred in low-income and middle-income countries [1]. In China, a developing country, injury has replaced diseases as the leading cause of death among elementary and secondary school students since the 1990s [6,7]. In particular, as road transport infrastructure developed, the incidence of road traffic injuries grew unexpectedly rapidly [8].

Injuries are a considerable threat to the health of children and adolescents and cause substantial economic loss for families and society [9]. The number of deaths of children from unintentional injuries is staggering. In the United States, more than 9,000 children die from unintentional injuries each year—about 25 deaths per day [10]. Non-fatal injuries may cause lifelong disability or chronic pain that limits the performance of daily activities. About 9 million children are treated for injuries in emergency departments yearly and more than 225,000 require hospitalization, incurring costs of around 87 billion USD in medical and societal expenses related to childhood injuries [5]. Additionally, the psychological burden caused by a family’s loss of a child is immeasurable.

Considerable evidence demonstrates that effective interventions can prevent the majority of childhood injuries. For example, setting minimum drinking age laws, lowering the blood alcohol concentration limit for drivers and reducing driving speeds around schools can effectively reduce childhood road traffic injuries [1,11]. A recent systematic review [12] indicated that prevention programs caused a significant reduction in adolescent injuries (relative risk (RR) = 0.62, 95% confidence interval (CI) [0.48, 0.81]), especially in girls. Both pre-season and in-season interventions were beneficial in reducing adolescent injuries. Another study showed that the average incidence of injury among secondary students reduced from 5.19% to 1.91% after a 5-month education and training program in Guangdong, China [13].

Because of the substantial epidemiological and economic burden of childhood injuries and because injuries are preventable, a study on the epidemiological characteristics and risk factors of childhood injury was necessary. While there were previous studies on this topic in China focusing on specific regions and populations, research on childhood and adolescent injuries in Xiamen, China had just begun and no relevant data sets were established. To provide a theoretical basis and empirical support for interventions, we aimed to (1) establish the prevalence and the major causes of injuries among elementary and secondary students in Xiamen, China; (2) identify the key indicators related to disparities in childhood and adolescent injuries; and (3) measure the economic and family burden of childhood and adolescent injuries. To accomplish these, we conducted an epidemiological study to understand the socio-demographic characteristics, risk factors and burden of injuries among elementary and secondary students in Xiamen, China.

## Methods

### Definition of injuries

In our study, we considered 14 types of injuries: traffic and road injuries, unintentional falls, collisions and strikes, sprains, cuts and sharp instrument injuries, crush injuries, drowning, suffocation, electrocution, burns and scalds, explosions, poisonings, animal attacks, and medical complications. Injury was defined following Wang SY (1998) [14] because the definition is suitable for China’s national condition and most commonly used in studies of injuries in China. An event is considered as injury if any of the following three events occurred: (a) being treated and diagnosed with injuries at a hospital; (b) receiving emergency treatment or care from family, teachers, or classmates; or (c) being absent from class for more than half a day because of injury.

### Sampling strategy

Subjects in our study were students in elementary (grades 1–6) and secondary (grades 7–12) schools in Xiamen, China. Multi-stage stratified cluster random sampling was performed to recruit students in 2010. Recruiting was performed by district. Xiamen is one of five special economic zones in China, and it has six districts: Huli, Siming, Haicang, Jimei, Xiang’an and Tong’an. According to economic conditions, the first two districts are classified as urban, the middle two districts as suburban, and the last two districts as exurban. At the first sampling stage, one urban, suburban, and exurban district was randomly selected. At the second stage, two elementary schools and two secondary schools were randomly selected from each selected district. At the third and last stage, we randomly chose one class from each grade in each school. Randomization was performed using simple random sampling in SPSS 17.0. All of the students in the sampled classes were enrolled in our study. Participants received a paper questionnaire and were asked to bring it home for their parents or guardians to answer the questions.

### Measures

The content of the questionnaire included the following. (1) Students’ basic information: sex, age, height, weight, health status (good, fair, poor), personality (introversive, intermediate, extroversive), and number of siblings. (2) Students’ family background: parents’ age, education, occupations, marital status (married, divorced, separated, widowed), and incomes. (3) Occurrence of injuries in the past year: number of injuries, time and location of injury, injury type, events, and affected part of the body. (4) Burden of injuries: economic and family-related.

The economic burden of injuries was measured by direct economic burden, including medical and other expenses directly related to treatments for injuries; indirect economic burden, which refers specifically to family income loss due to parents’ or families’ absence at work caused by the children’s injuries; and intangible economic burden, measured by the amount of money the family is willing to pay to avoid injuries. The family’s burden of injuries was measured using the Family Burden Scale of Diseases (FBS) [15], which includes six dimensions: financial burden (FM1), disruption of family routine activities (FM2), disruption of family leisure (FM3), disruption of family interaction (FM4), effect on physical health (FM5), and effect on mental health (FM6). Each dimension contains two to six items, and each item has three responses: no impact, moderate impact and serious impact, scoring 0, 1 and 2, respectively.

### Ethics statement

Informed consent was acquired on the first page of the questionnaire. All respondents participated voluntarily and provided informed consent. This study was approved by the ethical review committee of the School of Public Health, Xiamen University.

### Data analysis

A chi-square test was used to analyze the association between the incidence of injury and impact factors. A multivariate logistic regression model was used to analyze significant variables from the chi-square test. Cronbach’s *α* coefficient was used to measure the internal consistency and reliability of the FBS. The six FBS dimensions were assessed as follows: (1) calculate the dimension scores by averaging the item scores within each dimension for each student; and (2) obtain the thresholds for each dimension by averaging all the students’ dimension scores. There was considered to be no impact if the dimension score for an individual student is 0, moderate impact if larger than 0 but less than the threshold, and serious impact at or above the threshold. We summarize the distributions of each dimension by reporting the frequency and relative frequency of each response. All statistical analyses were conducted using SPSS 17.0. Data was double-entered and validated by two trained research assistants using Epidata 3.1.

## Results

### Sampling characteristics

A total of 2,960 questionnaires were issued and 2,918 finished questionnaires were received. The response rate was 98.6%. Of the finished questionnaires, 2,816 (96.5%) were complete therefore usable. Table 1 summarizes the demographic characteristics of the 2,816 students, their incidence of injuries, and the results of chi-square tests. In total, 303 students suffered from 365 injuries within the year, giving a 10.8% incidence of injuries. Boys were more likely to suffer injuries than girls. Students who were in grades 4–9, were living in suburban or exurban environments, were extroversive, had sibling(s), were of poor health status, were living with more than 7 people, or had a household income greater than 4,000 RMB/month had a higher risk of injuries. Students whose parent(s) were low-educated (primary educated or below), performed manual labor, or were unmarried (divorced, separated or widowed) were more likely to suffer from injuries. Notably, the incidence of injuries was higher among extroversive students than introversive ones. Of the 303 injured students, most (84.2%) were injured once, were non-introversive (94.4%), and were not overweight (86.9%); more than half were boys (58.7%), more than half were in grades 4–9 (58.4%), nearly three quarters (74.3%) lived in suburban or exurban environments, half (50.8%) were from a one-child family, and 60% had a household income of less than 4,000 RMB/month.

**Table 1:** **Demographic characteristics and chi-square test results for 2,816 participant students**

**Characteristic**	**All students**	**Injured students (times)**	**Incidence (times) (%)**	***χ*** ^***2***^	***P***
**Sex**				12.702	<0.001
Boys	1382	178 (216)	12.9 (15.6)		
Girls	1434	125 (149)	8.7 (10.4)		
**Grade**				12.617	0.006
1–3	737	61 (67)	8.3 (9.1)		
4–6	723	81 (100)	11.2 (13.8)		
7–9	691	96 (117)	13.9 (16.9)		
10–12	665	65 (81)	9.8 (12.2)		
**Residence**				8.854	0.012
Urban	939	78 (90)	8.3 (9.6)		
Suburban	918	109 (138)	11.9 (15.0)		
Exurban	959	116 (137)	12.1 (14.3)		
**BMI**				0.005	0.942
<24	2448	263 (316)	10.7 (12.9)		
≥24	368	40 (49)	10.9 (13.3)		
**Personality**				14.314	0.001
Introversive	282	17 (18)	6.0(6.4)		
Intermediate	1543	154 (153)	10.0(9.9)		
Extroversive	991	132 (194)	13.3(19.6)		
**Number of sibling**				7.011	0.008
0	1631	154 (182)	9.4(11.2)		
≥1	1185	149(183)	12.6(15.4)		
**Health Status**				13.453	0.001
Good	2144	211(253)	9.8(11.8)		
Fair	637	83(100)	13.0(15.7)		
Poor	35	17(12)	25.7(34.3)		
**Number of people living together**				9.772	0.008
≤3	1142	117(143)	10.2(12.5)		
4–6	1613	172(204)	10.7(12.6)		
≥7	61	14(18)	23.0(29.5)		
**Father’s educational level**				6.110	0.047
≤ grade 6	402	51 (61)	12.7(15.2)		
grade 7–12	1563	179(219)	11.5(14.0)		
> grade 12	831	72 (84)	8.7(10.1)		
Missing	20	1 (1)	---		
**Mather’s educational level**				8.404	0.015
≤ grade 6	824	109(140)	13.2(17.0)		
grade 7–12	1402	144(153)	10.3(10.9)		
> grade 12	582	50(72)	8.6(12.4)		
Missing	8	0	---		
**Father’s Occupation**				10.839	0.028
Farmer	175	16(21)	9.1(12.0)		
Manual labor	592	83(101)	14.0(17.6)		
White collar	999	94(110)	9.4(11.0)		
Individual merchant	636	74(89)	11.6(14.0)		
Others	394	35(43)	8.9(10.9)		
Missing	20	1(1)	---		
**Mother’s Occupation**				10.581	0.032
Farmer	138	12(16)	8.7(11.6)		
Manual labor	561	76(87)	13.5(15.5)		
White collar	876	77(92)	8.8(10.5)		
Individual merchant	440	56(63)	12.7(14.3)		
Others	793	82(107)	10.3(13.5)		
Missing	8	0			
**Parents’ marital status**				9.499	0.002
Married	2697	280(341)	10.4(12.6)		
Non-married	119	23(24)	19.3(20.2)		
Household income*				4.390	0.111
<2000	855	91 (107)	10.6(12.5)		
2000–3999	920	91 (106)	9.9(11.5)		
≥4000	719	94 (117)	13.1(16.3)		
Missing	322	27 (35)	---		
**Total**	2861	303 (365)	10.6(12.8)		

*RMB/month.

The characteristics of injury events are shown in Table 2. Here we group the months into four seasons because of seasonal variation in outdoor and indoor activities. We used morning, afternoon and evening as the three conventional time periods of the day. Of the 365 injuries, most (97.0%) were unintentional, more than one third occurred during the summer (June to August) (36.4%) and in school (35.1%), and more than half happened in the afternoon (55.3%) and affected the limbs (53.2%). The main causes of injury were unintentional falls (30.7%), collisions or strikes (18.8%), sprains (17.3%), and cuts or sharp instrument injuries (10.1%).

**Table 2:** **Characteristics of student injury incidents by proportion and percentage**

	**Number of injury**	**Proportion (%)**
**Type of injury**		
Unintentional	354	97.0
Intentional	11	3.0
**Month of injury**		
December to February	58	15.9
March to May	80	21.9
June to August	133	36.4
September to November	94	25.8
**Time of day**		
Morning	78	21.4
Afternoon	202	55.3
Evening	85	23.3
**Location**		
School	128	35.1
Home/Dormitory	76	20.8
Road/Street	63	17.3
Public places	47	11.5
Sports venues outside school	42	12.9
Other	9	2.5
**Part of body affected**		
Lower limb	113	31.0
Upper limb	81	22.2
Fingers/Toes	67	18.4
Face	37	10.1
Head and Neck	30	8.2
Torso	13	3.6
Multiple parts	13	3.6
Organ	11	3.0
**Total**	365	100.0

### Statistical analysis of injury impact factors

As Table 1 indicates, the significant impact factors for the incidence of injuries include sex, grade, residence, personality, number of siblings, health status, number of people living together, parents’ educational level, parents’ occupation, and parents’ marital status. Treating these impact factors as covariates, multivariate stepwise logistic regression was performed, and the results are presented in Table 3. The variables of parents’ education and parents’ occupation did not enter the model. Among the entered variables, sex, grade, residence, health status, personality, number of siblings, and parents’ marital status were statistically significant. The odds of injury were 1.8 times higher for boys than girls, 1.6 times higher for students living in exurban than in urban environments, 1.4 times higher for students in grades 4–9 than in grades 1–3, 2.9 times greater for extroversive students than introversive ones, 1.6 times greater for students with siblings than single children, and 2.8 times greater for students whose parents were of unmarried rather than married.

**Table 3:** **Results of multivariate stepwise logistic regression for injury impact factors**

**Factors**	***β***	**SE**	***Wald χ*** ^**2**^	***P***	***OR*** **(95% CI)**
**Sex**					
Girls					1.000
Boys	0.564	0.130	18.817	0.000	1.758 (1.362–2.268)
**Grade**					
1–3					1.000
4–6	0.309	0.182	2.875	0.090	1.363 (0.953–1.948)
7–9	0.368	0.184	4.021	0.045	1.445 (1.008–2.070)
10–12	−0.096	0.200	0.230	0.632	0.908 (0.613–1.345)
**Residence**					
Urban					1.000
Suburban	0.385	0.161	5.741	0.017	1.470 (1.073–2.014)
Exurban	0.486	0.159	9.312	0.002	1.626 (1.190–2.221)
**Health Status**					
Good					1.000
Fair	0.394	0.145	7.388	0.007	1.483 (1.116–1.971)
Bad	1.254	0.414	9.174	0.002	3.505 (1.557–7.892)
**Personality**					
Introvert					1.000
Middle	0.676	0.269	6.296	0.012	1.966 (1.159–3.334)
Extrovert	1.056	0.273	14.923	0.000	2.876 (1.683–4.915)
**Number of siblings**					
≥1					1.000
0	0.473	0.163	8.427	0.004	1.605 (1.166–2.210)
**Parents’ marriage**					
In marriage					1.000
Non-married	1.044	0.264	15.600	0.000	2.842 (1.692–4.771)
**Number of people living together**					
≤3					1.000
4–6	−0.166	0.159	1.083	0.298	0.847 (0.620–1.158)
≥7	0.734	0.343	4.568	0.033	2.083 (1.063–4.082)

### Economic burden of injuries

The direct economic burden of the 365 injuries among 303 students was 258,219.6 RMB (37,834.37 USD) total, 852.2 RMB (124.86 USD) per capita, and 707.5 RMB (103.66 USD) per incident. The outpatient expenses were 91,396.6 RMB (13,391.44 USD) (35.4%) and the hospitalization expenses were 147,885 RMB (21,668.13 USD) (57.3%). The expenses for travel, care, nutrition and rehabilitation supplies were 18,938 RMB (2,774.80 USD) (7.3%). The indirect economic burden of the 365 injuries was 88,948RMB (13,032.67 USD) total, 293.6 RMB (43.02 USD) per capita, and 243.7 RMB (35.71 USD) per incident. The intangible economic burden was 667,481.5 RMB (97,799.49 USD) total, 2,202.9 RMB (322.77 USD) per capita, and 1,828.7 RMB (267.94 USD) per incident. Altogether, the total economic burden (including the direct, indirect, and intangible economic burden) of the 365 injuries amounted to 1,014,649.1 RMB (148,666.5 USD) total, 3,348.7 RMB (490.65 USD) per capita, and 2,779.9 RMB (407.31 USD) per incident.

The economic burden of injuries with different characteristics is presented in Table 4. Although the incidence of injuries in students in grades 10–12 was lower, the direct and intangible economic burdens (1,146.3 RMB (167.96 USD) and 2,932.1 RMB (429.61 USD), respectively) associated with their injuries were higher. The indirect economic burden for students in grades 1–3 was higher (340.3 RMB (49.86 USD)). The total economic burden was higher (4,175.9 RMB (611.85 USD)) for students living in urban areas. Among the 14 causes of injury, the highest per capita economic burdens were caused by medical complications, poisoning, and traffic accidents.

**Table 4:** **Average direct, indirect, intangible and total financial burden of student injuries per person-time (RMB) (1 RMB = 0.1465 USD)**

	**Person-time**	**Economic burden**			**Total**
		**Direct**	**Indirect**	**Intangible**	
**Sex**					
Boys	216	751.6	239.7	1823.1	2814.4
Girls	149	643.5	249.5	1836.8	2729.8
**Grade**					
1–3	67	854.2	340.3	2480.6	3675.1
4–6	100	556.9	197.2	1443.8	2197.9
7–9	117	448.3	244.7	1020.6	1713.6
10–12	81	1146.3	219.8	2932.1	4298.1
**Residence**					
Urban	90	1056.5	486.7	2632.7	4175.9
Suburban	138	593.1	162.7	1460.6	2216.3
Exurban	137	593.3	165.7	1671.4	2430.4
**Causes**					
Unintentional falls	112	724.4	340.6	1520.9	2586.0
Collisions/Strikes	68	326.3	294.4	1347.0	1967.7
Sprains	63	311.4	83.0	592.5	986.9
Cuts/Sharp instrument	37	93.6	66.2	152.4	312.3
Burns and scalds	21	347.5	128.6	948.0	1424.1
Traffic accidents	20	2300.9	525.0	6685.0	9510.9
Animal attacks	14	210.9	39.3	357.9	608.0
Crush	13	283.1	57.7	631.5	972.3
Medical complications	8	7688.1	625.0	17506.3	25819.4
Explosion	4	287.5	125.0	500.0	912.5
Poisonings	3	2066.7	1033.3	16900.0	20000.0
Others	2	1502.5	0.0	1500.0	3002.5
**Total**	365	707.5	243.7	1828.7	2779.9

Table 5 presents the frequencies and relative frequencies of different responses to the six dimensions of FMS. The positive response rates (moderate impact and serious impact) rates were: financial burden (FM1) (58.8%), the disruption of family routine activities (FM2) (65%), the disruption of family leisure (FM3) (58.4%), and the disruption of family interaction (FM4) (46.2%). For the FMS overall, the positive response rate was 74.9%. The Cronbach *α* for the six dimensions (FM1–FM6) were 0.889, 0.912, 0.907, 0.926, 0.902, and 0.892, respectively, and 0.961 for the FMS overall.

**Table 5:** **Family Burden Scale of Diseases (FBS) dimension measurements**

**Dimension**	**No impact**		**Moderate impact**		**Serious impact**	
	***n***	**Ratio (%)**	***n***	**Ratio (%)**	***n***	**Ratio (%)**
Financial burden (FM1)	125	41.3	46	15.2	132	43.6
Disruption of family routine activities (FM2)	106	35.0	56	18.5	141	46.5
Disruption of family leisure (FM3)	126	41.6	46	15.2	131	43.2
Disruption of family interaction (FM4)	163	53.8	30	9.9	110	36.3
Effect on Physical health (FM5)	215	71.0	0	0.0	88	29.0
Effect on Mental health (FM6)	221	72.9	0	0.0	82	27.1
The entire scale (FM)	76	25.1	109	36.0	118	38.9

## Discussion

Injury has become a public health problem that threatens elementary and secondary students’ health and causes substantial economic burden. Our study shows that the incidence of injuries in elementary and secondary students in Xiamen, China was 10.8%, lower than in Guangdong and Zhejiang provinces [16-20], but higher than in Beijing and Shanghai [21,22]. Extrapolating from data from the Xiamen Special Economic Zone Yearbook of 2013 [23], we estimate that 45,361 injuries have occurred annually among 37,685 elementary and secondary students in Xiamen, given that there were a total of 348,933 elementary and secondary students in Xiamen in 2012.

The incidence of injuries for the students living in suburban areas (12.0%) was higher than those in urban areas (8.3%). This may be because urban students had better living and learning environments and were well supervised and protected by their guardians. The incidence of injuries among boys (12.9%) was higher than girls (8.7%), which is consistent with other regional research results [16-22]. Boys were more exposed to risk factors because they were more likely to join in outdoor activities like sports, while girls were less likely to undergo injuries because they were relatively cautious and introversive.

In addition, students in grades 4–9 were more likely to suffer injuries than those in grades 1–3 or 10–12. One possibility is that the students in grades 4–9 were more playful but lacked the self-awareness of protection, while the younger students were closely supervised by both teachers and parents, and the older students were too busy with their courses and preparing for the college entrance examination to participate in outdoor activities. Furthermore, the senior students were less likely to get injured because they had better physical coordination and greater awareness of self-protection. However, when injuries occurred, the economic burden for older students was higher, which indicates that their injuries were more serious. Students whose parents’ educational levels were lower had a higher risk of injuries. Parents have an integral role in their children’s growth, and in general, more highly educated parents provide better education and supervision for their children to avoid injuries.

Almost all (97.0%) injuries were unintentional. More than one third of the students’ injuries occurred from June to August, likely because students engaged in outdoor activities more frequently in summer, with less protection from clothing and with more of their body exposed. In addition, more than half of the injuries occurred in the afternoon, the peak time for outdoor sports activities.

Most injuries of elementary and secondary school students occurred at school (35.1%), at home or in dormitories (20.8%), and on the road (17.3%), which were the major places where students spent their time. In all areas and grades investigated, the school was where most injury occurred, so it is essential that schools provide a safe environment with high-quality education and living facilities to prevent and control student injuries. Arms/legs and fingers/toes were the main affected body parts, which relates to the top three causes of injury: unintentional falls (30.7%), collisions/strikes (18.8%), and sprains (17.3%).

Multivariate stepwise logistic regression analysis showed that the incidence of injuries varied significantly among areas, sexes and grades. The results suggest that injury prevention and control interventions should focus on male elementary and secondary school students in suburban and exurban areas. Second, student injuries varied by health condition and personality. Students in fair or poor health were more likely to be affected by various levels and types of injuries. Extroversive students had more exposure to injury risk factors because they were more physically active. Therefore, we suggest that the primary aim of injury prevention and control interventions be to strengthen students’ physique, cultivate their safety awareness, and improve their self-protection abilities. Third, students’ injuries might be caused by their domestic situation. A child with sibling(s) may receive less concern and care from parents compared with a single child. Consequently, a child with sibling(s) is more likely to be ignored, and their risk of injury is accordingly higher. The odds of injury were higher in single-parent families. In addition, more people living together increased the chances of friction between students and others, thereby increasing the risk of injuries.

As our study indicated, the direct economic burden of student injury was 258,219.6 RMB (37,834.37 USD) in total, 852.2 RMB (124.86 USD) per capita and 707.5 RMB (103.66 USD) per incident from 365 injury incidents in 303 elementary and secondary school students in Xiamen in one year. This economic burden was higher than in Haidian District and Pinggu District in Beijing [21]. This might be because the Beijing survey was conducted earlier and the inflation rate was relatively high in recent years. In addition, injuries caused a total indirect economic burden of 88,948 RMB (13,032.67 USD) and a total intangible economic burden of 66,7481.5 RMB (97,799.49 USD). We estimate that injuries in elementary and secondary school students in Xiamen cause a direct economic burden of 27.42 million RMB (4.02 million USD), an indirect economic burden of 9.44 million RMB (1.38 million USD), an intangible economic burden of 70.87 million RMB (10.38 million USD), and a total economic burden of 108 million RMB (15.82 million USD) each year. We must take effective measures to control and reduce the occurrence of injuries because of the serious economic burden of student injuries on individuals, families, and society.

This study found that the intangible economic burden of injury accounted for nearly 70% of the total economic burden. Injuries not only wasted money for individuals, families, and society but also were a burden on the emotional wellbeing of individuals and families. Because elementary and secondary students are the hope of each family and of society in general, the psychological loss for families caused by injuries is substantial. Therefore, the overall costs saved for society by effective prevention and control measures to reduce the occurrence of injuries in elementary and secondary students will be far greater than the actual material goods and money saved on treatment.

Injuries cause both the burden of disease and severe psychological pressure and life consequences for members of the family. The impacts of injuries on elementary and secondary school students are pervasive and multi-dimensional. Injuries are undoubtedly preventable with appropriate prevention and control measures. According to our research results, we provide the following suggestions for the government, the local Centers for Disease Control and Prevention (CDC), schools and families to develop effective measures to control the incidence of injuries.

First, the government should focus and invest more on injury control and prevention within the school sector, especially in suburban and exurban areas. Secondly, schools should practice safety management strictly and do their best to eliminate the risk factors of injuries. Third, besides helping students to obtain academic achievements, schools should work together with parents on strengthening student physique, cultivating safety awareness, and improving self-protection abilities. For instance, Bjӧrklund et al. introduced a school-based intervention program, “Together at school”, in Finland to promote elementary school students’ socio-emotional skills and mental health [24]. Finally, the local CDC should collaborate with schools to establish a system for monitoring the incidence and prevalence of injuries to collect relevant information to provide theoretical and empirical support for the prevention programs [25]. Injury control and prevention among elementary and secondary school students is essential and will help in multiple ways to reduce the burden on the family to build a harmonious family and society.

Because of time and man-power limitations, our study collected fewer than 3,000 usable questionnaires and conducted analyses based on these data. Therefore, our results might not be representative of all the elementary and secondary school students in Xiamen, China. Although the randomly chosen schools appared to be representative and we do not believe that the sample size has biased our results, closer investigation about their representation needs to be done in the future. We were aware of this limitation, and we encourage scholars and practitioners to conduct more extensive surveys to obtain more representative results.

## Conclusion

The injury incidence among elementary and secondary students in Xiamen is lower than Guangdong and Zhejiang but high er than Beijing and Shanghai. Injuries cause enormous economic and familial burdens and threaten students’ health and life. Childhood and adolescent injuries have become a serious public health problem and require the urgent attention of the government, society, schools, and families.
